# OpenCOR: a modular and interoperable approach to computational biology

**DOI:** 10.3389/fphys.2015.00026

**Published:** 2015-02-06

**Authors:** Alan Garny, Peter J. Hunter

**Affiliations:** Auckland Bioengineering Institute, The University of AucklandAuckland, New Zealand

**Keywords:** computational biology, software, interoperability, CellML, metadata

## Abstract

Computational biologists have been developing standards and formats for nearly two decades, with the aim of easing the description and exchange of experimental data, mathematical models, simulation experiments, etc. One of those efforts is CellML (cellml.org), an XML-based markup language for the encoding of mathematical models. Early CellML-based environments include COR and OpenCell. However, both of those tools have limitations and were eventually replaced with OpenCOR (opencor.ws). OpenCOR is an open source modeling environment that is supported on Windows, Linux and OS X. It relies on a modular approach, which means that all of its features come in the form of plugins. Those plugins can be used to organize, edit, simulate and analyze models encoded in the CellML format. We start with an introduction to CellML and two of its early adopters, which limitations eventually led to the development of OpenCOR. We then go onto describing the general philosophy behind OpenCOR, as well as describing its openness and its development process. Next, we illustrate various aspects of OpenCOR, such as its user interface and some of the plugins that come bundled with it (e.g., its editing and simulation plugins). Finally, we discuss some of the advantages and limitations of OpenCOR before drawing some concluding remarks.

## Introduction

Traditionally, the development of a mathematical model starts by laying down some ideas on paper. These ideas then get implemented in some programming language, such as C++, MATLAB or Python. A few iterations between paper and coding are usually required to get a working model. Once this is done, the model is shared with the community, which is typically done by writing and submitting a manuscript that describes the model, and includes its equations, initial conditions, etc. Upon successful peer-review, the manuscript is published, making it possible for interested parties to implement and use the model (Garny et al., 2009).

However, such an approach is error prone. Errors can be introduced at every stage (e.g., during the publishing process, during the implementation of a model by an interested party). For this reason and others (e.g., the reproducibility of published simulation results), a lot of effort has been put into the development of standards and formats, with the intention of making it easier to describe and exchange experimental data, mathematical models, simulation experiments, etc. Such efforts include CellML (cellml.org; Cuellar et al., 2003), SBML (sbml.org; Hucka et al., 2003) and SED-ML (sed-ml.org; Waltemath et al., 2011), all of which are now coordinated under the COMBINE initiative (co.mbine.org), as discussed in Hucka et al. (2015).

CellML is a format for describing and exchanging biological models. It is based on XML (w3.org/XML; Bray et al., 2000) and relies on MathML (w3.org/Math; Ausbrooks et al., 2001) for describing the mathematics and RDF/XML (w3.org/TR/REC-rdf-syntax) for annotations. It is primarily used in computational biology to encode and annotate systems of ordinary differential equations (ODEs), as well as of differential algebraic equations (DAEs). CellML was first released in August 2001 (CellML 1.0) and refined in February 2006 (CellML 1.1) by allowing certain CellML concepts to be imported and reused.

COR (cor.physiol.ox.ac.uk) was the first publically available CellML-based environment (Garny et al., 2003, 2009). OpenCell (opencell.org), formerly known as PCEnv, came next. COR is still being actively used, but it is a native Windows application and it only supports CellML 1.0. OpenCell does not have those limitations, but unlike COR its authoring capabilities are limited. Also, it takes significantly longer to run simulations in OpenCell compared with COR (Garny et al., 2008). For those reasons and others, the two groups behind COR and OpenCell agreed to discontinue their respective efforts and, instead, collaborate on OpenCOR (opencor.ws).

OpenCOR is an open source environment that can be used to organize, edit, simulate and analyze models of ODEs or DAEs encoded in the CellML format. OpenCOR was built from the ground up, using a modular approach to make it easier to expand (unlike COR and OpenCell). It includes the features that have made COR and OpenCell successful (e.g., their simulation capabilities), as well as new ones (e.g., its annotation capabilities), and works on Windows, Linux and OS X.

This technology report starts with a description of the general philosophy behind OpenCOR (Section General Philosophy), followed by some examples of its openness (Section Openness) and some information on its development process (Section Software Development). Several aspects of OpenCOR are then illustrated, including its user interface (Section User Interfaces), its help plugin (Section Help Plugin), its organization plugins (Section Organization Plugins), its editing plugins (Section Editing Plugins), its simulation plugin (Section Simulation Plugin) and its solver plugins (Section Solver Plugins). Some discussions on programming languages and frameworks (Section Programming Languages and Frameworks), the CellML API^1^ (Section CellML API), the *Editing* mode (Section Editing Mode), the *Simulation* mode (Section Simulation Mode), other standards (Section Other Standards) and other CellML tools (Section Other CellML Tools) come next, completed by some concluding remarks (Section Conclusion).

## Materials and methods

### General philosophy

From the outset, OpenCOR was designed to be a modeling environment that can be used to organize, edit, simulate and analyze mathematical models. Initial support for those models was to come through CellML. However, OpenCOR had to be modular, so that support for other standards and formats (e.g., from the COMBINE initiative: SBML, SED-ML, etc.) could be added to it. This modularity was also to be used to add new capabilities to OpenCOR (e.g., a new way to edit models, a new numerical solver). Finally, OpenCOR was to be usable both from the command line and from a GUI, and this on Windows, Linux and OS X.

### Openness

#### Open source license

OpenCOR was always intended to be an open source project, but the license under which it was to be released had to be approved by the Open Source Initiative (OSI; opensource.org), be business friendly, offer patent protection, have no copyleft requirements, and be compatible with major OSI licenses. Apache 2.0 (opensource.org/licenses/Apache-2.0) is one such license and it is the license under which OpenCOR is released.

#### Online presence

As a project, OpenCOR tries to be as transparent as possible through its online presence. Its source code is thus available on GitHub (github.com) at github.com/opencor/opencor, which is also where all current (and former) bugs, feature requests, etc. can be found (github.com/opencor/opencor/issues).

As part of its software development process (see Subsection Codebase), OpenCOR is regularly built and tested on Linux and OS X, using the free service provided by Travis CI (travis-ci.org). The outcome of those builds and tests is available online at travis-ci.org/opencor/opencor.

OpenCOR has its own website (opencor.ws). It consists of a downloads section, which includes both the official releases and the recent snapshots of OpenCOR (opencor.ws/downloads). It also consists of a copy of both the user and developer documentations (opencor.ws/user and opencor.ws/developer, respectively). Except for one noticeable feature (see Section Help plugin), both the online and the embedded (in OpenCOR) versions of the user documentation are the same. The developer documentation is only available online, though it can also be found in the OpenCOR GitHub repository (as can the rest of the website contents).

### Software Development

#### Prerequisites

The OpenCOR project is set up in such a way that only Git (git-scm.com), CMake (cmake.org), a C++ toolchain and the Qt framework (qt.io) are needed to build, test and run OpenCOR (opencor.ws/developer/buildTestAndRun.html), and to package it (opencor.ws/developer/package.html). However, compilation can be sped up by using Ninja (martine.github.io/ninja), and additional packages can be generated by using NSIS (nsis.sourceforge.net) on Windows and PackageMaker on OS X (opencor.ws/developer/prerequisites.html).

#### Codebase

A specific file structure (opencor.ws/developer/fileStructure.html) is used to keep track of the various OpenCOR files. To make the OpenCOR source code as consistent and as maintainable as possible, a specific coding style is also followed (opencor.ws/developer/develop/codingStyle.html). Work on the OpenCOR codebase is referenced in GitHub issues (opencor.ws/developer/develop), which upon closing trigger a Travis CI job, to ensure that OpenCOR still builds and tests fine on both Linux and OS X (Travis CI does not currently support Windows). Depending on the work that has been done, a snapshot version of OpenCOR may also be released.

#### Modular approach

As a modular application, OpenCOR is effectively an empty shell, to which features are added by enabling one or several plugins (opencor.ws/developer/develop/plugins). To that end, OpenCOR supports various interfaces. For example, the GUI interface can be implemented by a plugin to let OpenCOR know about the menus and menu actions that the plugin wants to see added to the GUI, as well as for the plugin to be told whenever the GUI needs to be updated. By implementing one or several of those interfaces, it is possible to create different types of plugins, including multilingual ones (opencor.ws/developer/develop/internationalisation.html). Test-driven development (TDD) is also supported, at the plugin level (opencor.ws/developer/develop/tests.html), and plugins that implement TDD have their tests automatically run by Travis CI.

The developer documentation provides some information on how to develop plugins for OpenCOR. The *Sample* plugin (opencor.ws/developer/develop/plugins/Sample.html) is a non-selectable plugin (see Subsection Plugins window). It contains a simple add() function that can be used by other plugins, such as the *Sample tools* plugin (opencor.ws/developer/develop/plugins/SampleTools.html). That latter plugin makes it possible to add two numbers using either the CLI or the GUI version of OpenCOR. The *Sample window* plugin (opencor.ws/developer/develop/plugins/SampleWindow.html) implements another type of plugin by extending the *Sample* plugin and providing a dockable window to add two numbers. Finally, there is the *Sample view* plugin (opencor.ws/developer/develop/plugins/SampleView.html), which can be used to get some information on the current file (e.g., its SHA-1 value, its size).

#### Third-party libraries

OpenCOR makes use of the “official” CellML API (cellml-api.sourceforge.net; Miller et al., 2010) and several software- and image-based third-party libraries (opencor.ws/developer/thirdPartyLibraries.html). To build the CellML API requires additional build dependencies. As a consequence, it is not included in the OpenCOR build process. Instead, the CellML API is built on the side and its binaries are retrieved by CMake when building the *CellML API* plugin.

Software-based third-party libraries are either built as part of OpenCOR itself or as plugins, and are therefore included in the OpenCOR codebase. However, to save compilation time, CMake retrieves a pre-built version of the third-party plugins, although those can be individually included in the build process, if needed.

The third-party libraries built as plugins are LLVM (llvm.org), QScintilla (riverbankcomputing.co.uk/software/qscintilla) and SUNDIALS (computation.llnl.gov/casc/sundials; Hindmarsh et al., 2005). LLVM is a collection of modular and reusable compiler and toolchain technologies (it includes Clang, a compiler frontend to LLVM; see Section Simulation plugin), QScintilla an editing widget (see Subsections Raw view plugin and Raw CellML view plugin), Qwt a set of widgets (see Subsection Raw CellML view plugin and Section Simulation plugin), and SUNDIALS a suite of non-linear and differential/algebraic equation solvers (see Section Solver plugins).

## Results

### User Interfaces

OpenCOR can be run both as a CLI and a GUI application. However, so far, the focus has mainly been on its GUI version.

#### Command line interface

Besides typical CLI features (help, version and about information), the CLI version of OpenCOR can list the CLI plugins that are available (only the *CellML tools* plugin for now), as well as provide the status of all the plugins (i.e., the *CellML tools* plugin and all the other plugins on which it depends), as illustrated at opencor.ws/user/userInterfaces/commandLineInterface.html.

The *CellML tools* plugin works with both the CLI and GUI versions of OpenCOR (opencor.ws/user/plugins/miscellaneous/CellMLTools.html). It is used to export a CellML 1.1 file to CellML 1.0. It can also be used to export a CellML file to a user-defined format. Several such formats for C, FORTRAN 77, MATLAB and Python are shipped with OpenCOR.

#### Graphical user interface

**Figure 1:**
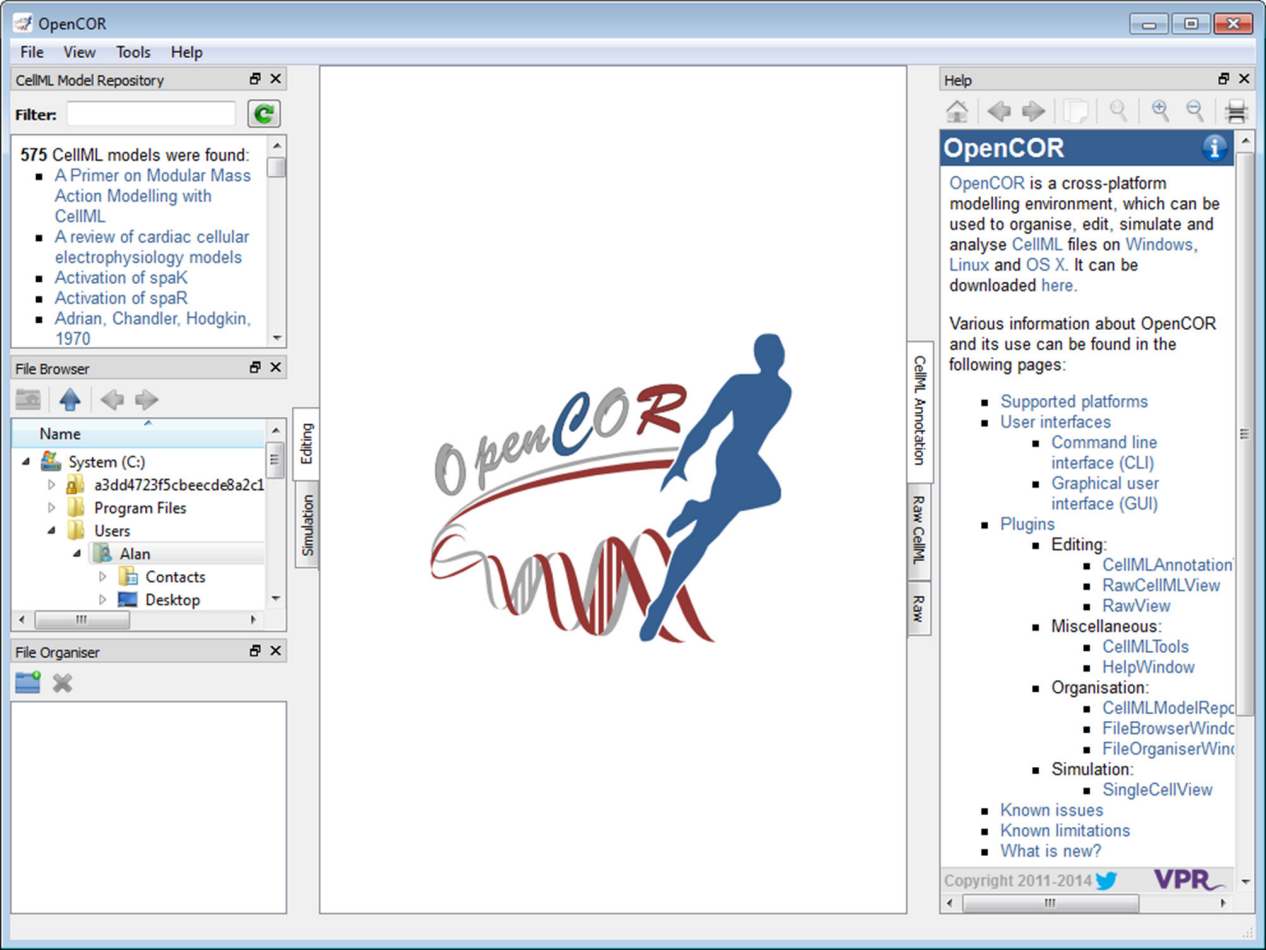
**Graphical user interface**. The graphical user interface consists of a central area where files are loaded and rendered (the OpenCOR logo is shown, if no files are opened). The rendering of a file depends on the selected view (to the right of the central area). In the case of the *Editing* mode (to the left of the central area), the views currently available are the *CellML Annotation* view, the *Raw* view and the *Raw CellML* view. Several windows (the *CellML Model Repository* window, the *File Browser* window, the *File Organiser* window and the *Help* window) are also available. By default, they are docked around the central area, but they can also be hidden or undocked.

Figure 1 shows what the GUI version of OpenCOR looks like when started for the very first time (opencor.ws/user/userInterfaces/graphicalUserInterface.html). Initially, no files are opened, so the OpenCOR logo is shown in the central area of the GUI. Upon opening a file, the logo is replaced with a rendering of the file. The rendering is based on the selected mode and view (to the left and to the right of the central area, respectively). There are currently two modes available: *Editing* and *Simulation*. Each mode offers one or several views. In the case of the *Editing* mode, those views are the *CellML Annotation* view, the *Raw* view and the *Raw CellML* view.

The four windows (the *CellML Model Repository*, the *File Browser*, the *File Organiser* and the *Help* windows) to the left and to the right of the central area can be docked anywhere around that area. Alternatively, they can be hidden or undocked.

#### Plugins window

**Figure 2:**
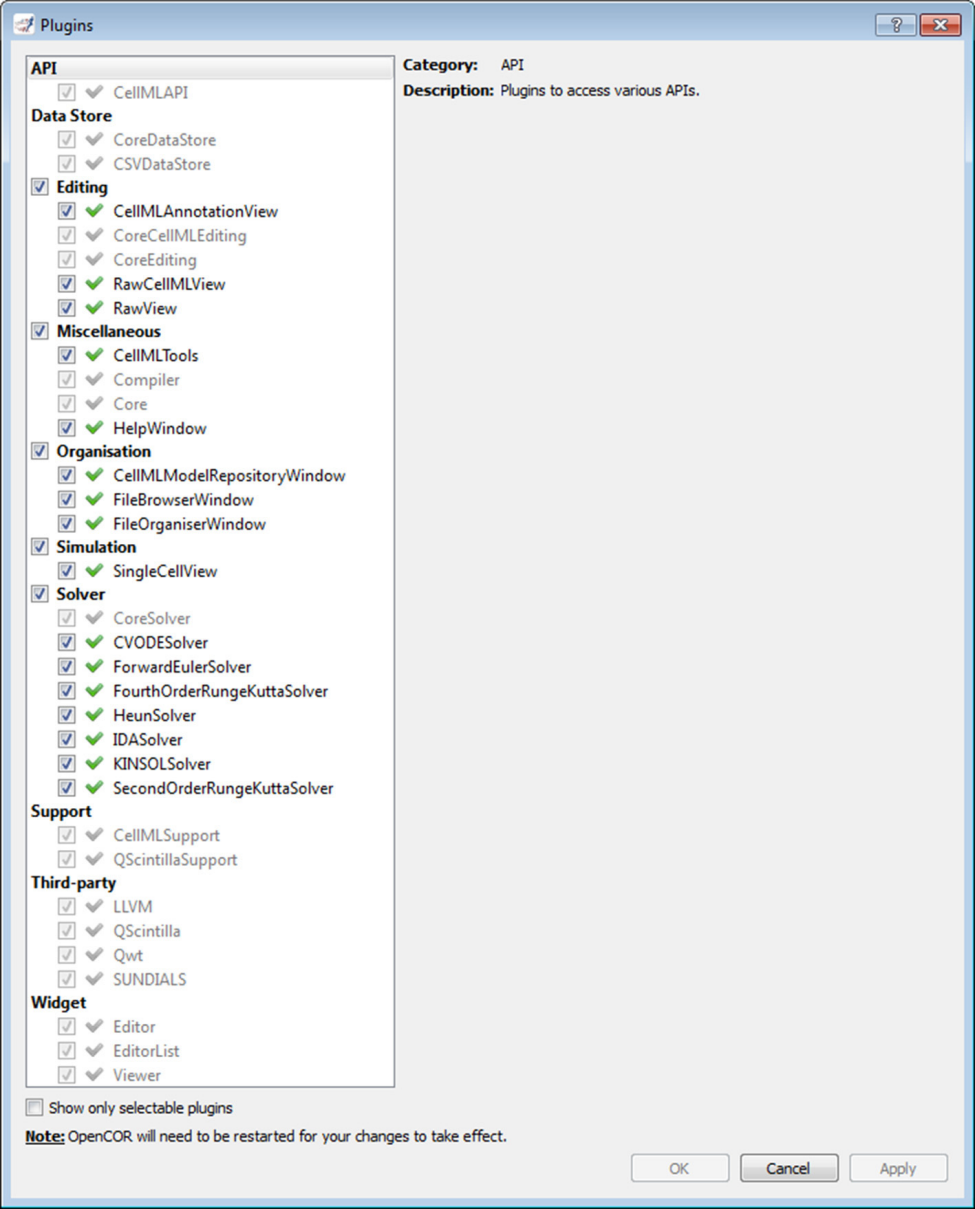
**Plugins window**. Several plugins are available, grouped in different categories. Grayed out plugins cannot be manually selected since they refer to a plugin that provides some functionality (e.g., CellML support), but that is of no use to OpenCOR on its own. Instead, they are automatically selected if they are needed by a selected selectable plugin.

As previously mentioned, every feature available in OpenCOR comes as a plugin. Figure 2 shows the *Plugins* window. It lists all the plugins available in OpenCOR, grouped in different categories for convenience. There are two types of plugins: selectable and non-selectable. Non-selectable plugins provide some functionality (e.g., CellML support), but they are of no use on their own. They can, however, be used by other plugins, be they selectable or not (opencor.ws/user/plugins).

### Help plugin

**Figure 3:**
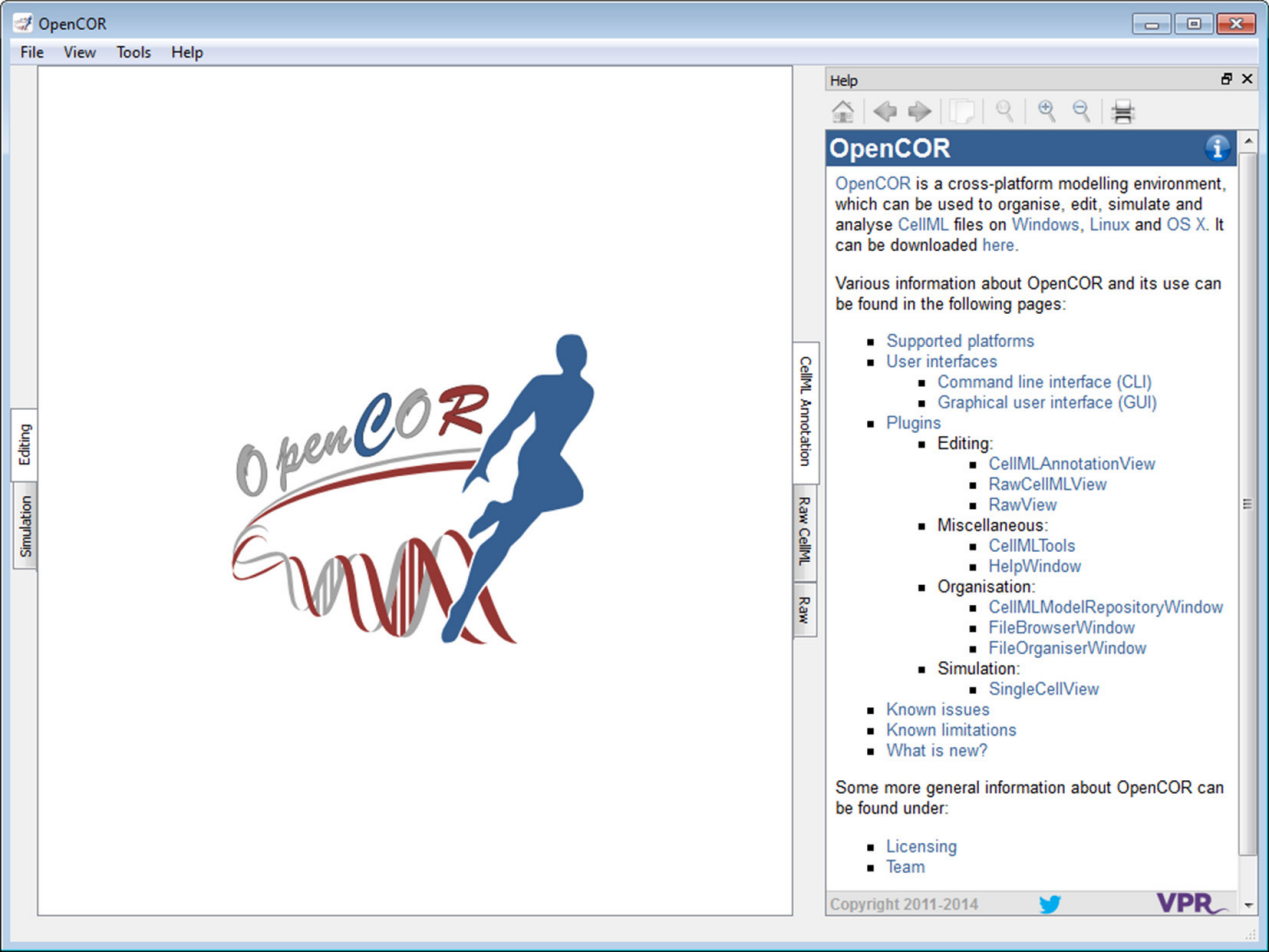
**Help window**. The *Help* window shows the contents of the online user documentation (opencor.ws/user).

The *Help window* plugin provides a dockable window that contains the user documentation (Figure 3; opencor.ws/user/plugins/miscellaneous/HelpWindow.html). Its contents is the same as the one that can be found online (opencor.ws/user). This includes a menu that gets shown whenever you move your mouse pointer over the information icon (top right of the *Help* window).

In addition to what is shown online, the *Help* window also displays special links, which when clicked send a command to OpenCOR. For example, one such link is used to open the *About* box. This feature has yet to be fully taken advantage of, but an area where it could be useful is the development of tutorials where similar links could be used to execute particular steps (e.g., to create a new CellML file, to start a simulation).

### Organization plugins

There are three organization plugins: the *CellML Model Repository window* plugin, the *File Browser window* plugin and the *File Organiser window* plugin (from top to bottom of the left hand side of Figure 1).

#### CellML Model Repository window plugin

The *CellML Model Repository window* plugin offers a simple interface to the CellML Model Repository (models.physiomeproject.org/cellml). By default, it lists all the CellML models found in the repository (opencor.ws/user/plugins/organisation/CellMLModelRepositoryWindow.html). However, the list can be filtered using either some plain text or a regular expression. To click on any of the listed links will open the workspace for that model in the user's default web browser. From there, the user can, for example, retrieve the latest exposure for that model.

#### File Browser window plugin

The *File Browser window* plugin provides a convenient way to access the user's physical files (opencor.ws/user/plugins/organisation/FileBrowserWindow.html). It starts with the user's home directory and, from one session to another, remembers the folder or file that was last selected. As can be expected, to double click on a folder will expand its contents while double clicking on a file will open it in OpenCOR. The rendering of the file will depend on the mode and view being currently selected. Files can be dragged from the *File Browser* window and dropped on to the *File Organiser* window.

#### File Organiser window plugin

The *File Organiser window* plugin allows a user to organize his/her files in a virtual manner, i.e., independently of where they are physically located (opencor.ws/user/plugins/organisation/FileOrganiserWindow.html). The virtual environment is remembered from one session to another and is originally empty. (Nested) virtual folders can be created to contain links to physical files. A virtual folder can be moved around, renamed or deleted. A link can also be deleted, if it is not needed anymore. Finally, in addition to using the *File Browser* window to drag and drop files on to the *File Organiser* window, any file manager can also be used for that purpose.

### Editing plugins

There are currently three view plugins that can be used to edit CellML and non-CellML files: the *CellML Annotation view* plugin, the *Raw view* plugin and the *Raw CellML view* plugin.

#### CellML Annotation view plugin

**Figure 4:**
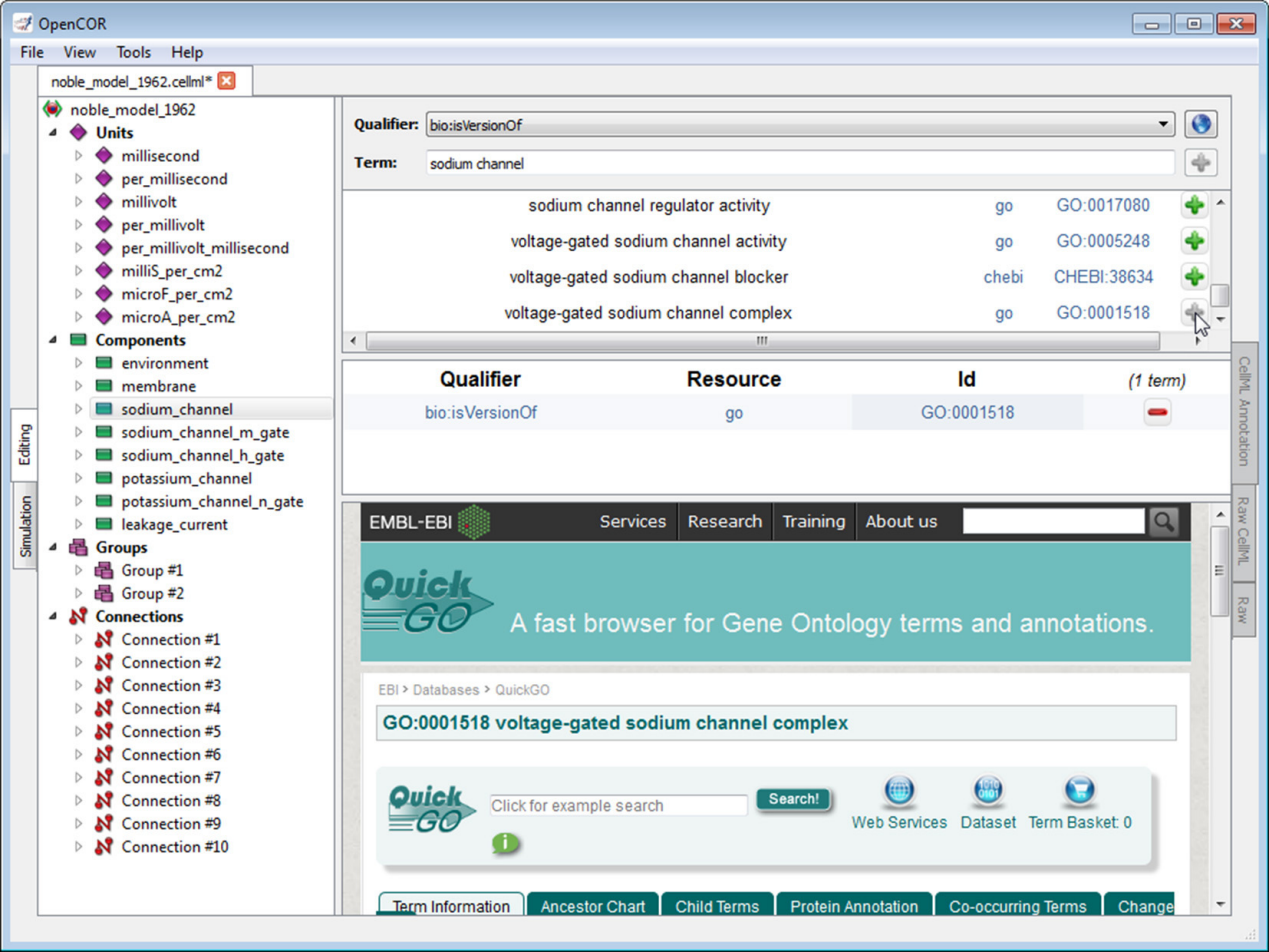
**CellML Annotation view**. The *CellML Annotation* view allows the annotation of CellML files using both BioModels.net qualifiers and ontological terms from different ontologies (e.g., CHEBI, FMA, GO).

The *CellML Annotation view* plugin is used to annotate CellML files (Figure 4; opencor.ws/user/plugins/editing/CellMLAnnotationView.html). The annotation is done at the CellML element level and consists of creating a clear relationship between a CellML element and a resource. A CellML element must be selected from the tree located to the left of the view. Next, to ensure the unambiguity of the relationship, we must select one of the several biomodels.net qualifiers (co.mbine.org/standards/qualifiers). Finally, a suitable resource needs to be used. In the context of computational biology, such a resource is understood to be a term from an established ontology (e.g., CHEBI, FMA, GO). However, there are thousands of ontological terms, so to help the user choose a suitable ontological term, a search term or a regular expression can be entered, for the view to use to retrieve a list of matching terms from different ontologies. The retrieval itself is done by sending a request to an instance of RICORDO (ricordo.eu; de Bono et al., 2011). Using that list, the user can decide which ontological term to use, after looking it up, if needed (as illustrated in Figure 4 by looking up the “voltage-gated sodium channel complex” term from the GO ontology). Annotations are stored using RDF triples (w3.org/TR/rdf11-concepts) with ontological terms in the form of identifiers.org URIs ^2^ (MIRIAM URNs ^3^ are also recognized, although they have now been deprecated in favor of identifiers.org URIs; Laibe and Le Novère, 2007; Juty et al., 2011).

#### Raw view plugin

**Figure 5:**
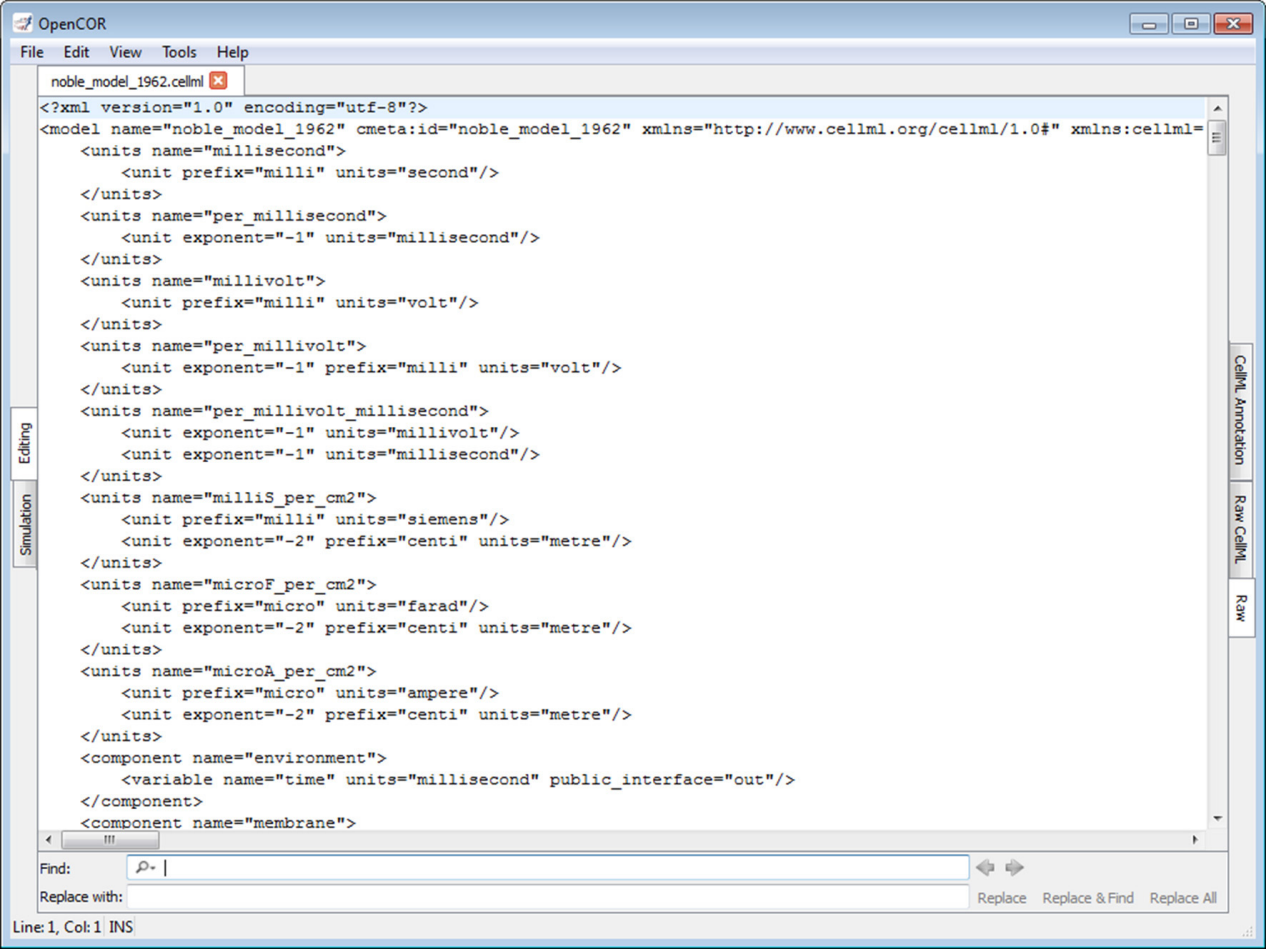
**Raw view**. The *Raw* view offers a way to edit files using a text-based editor.

The *Raw view* plugin allows the editing of text-based files using the QScintilla widget (Figure 5; opencor.ws/user/plugins/editing/RawView.html). Its font size can be increased and decreased, as needed, and traditional editing features, such as copying/pasting, undoing/redoing, etc. are available. A find/replace feature is also available, together with support for regular expressions.

#### Raw CellML view plugin

**Figure 6:**
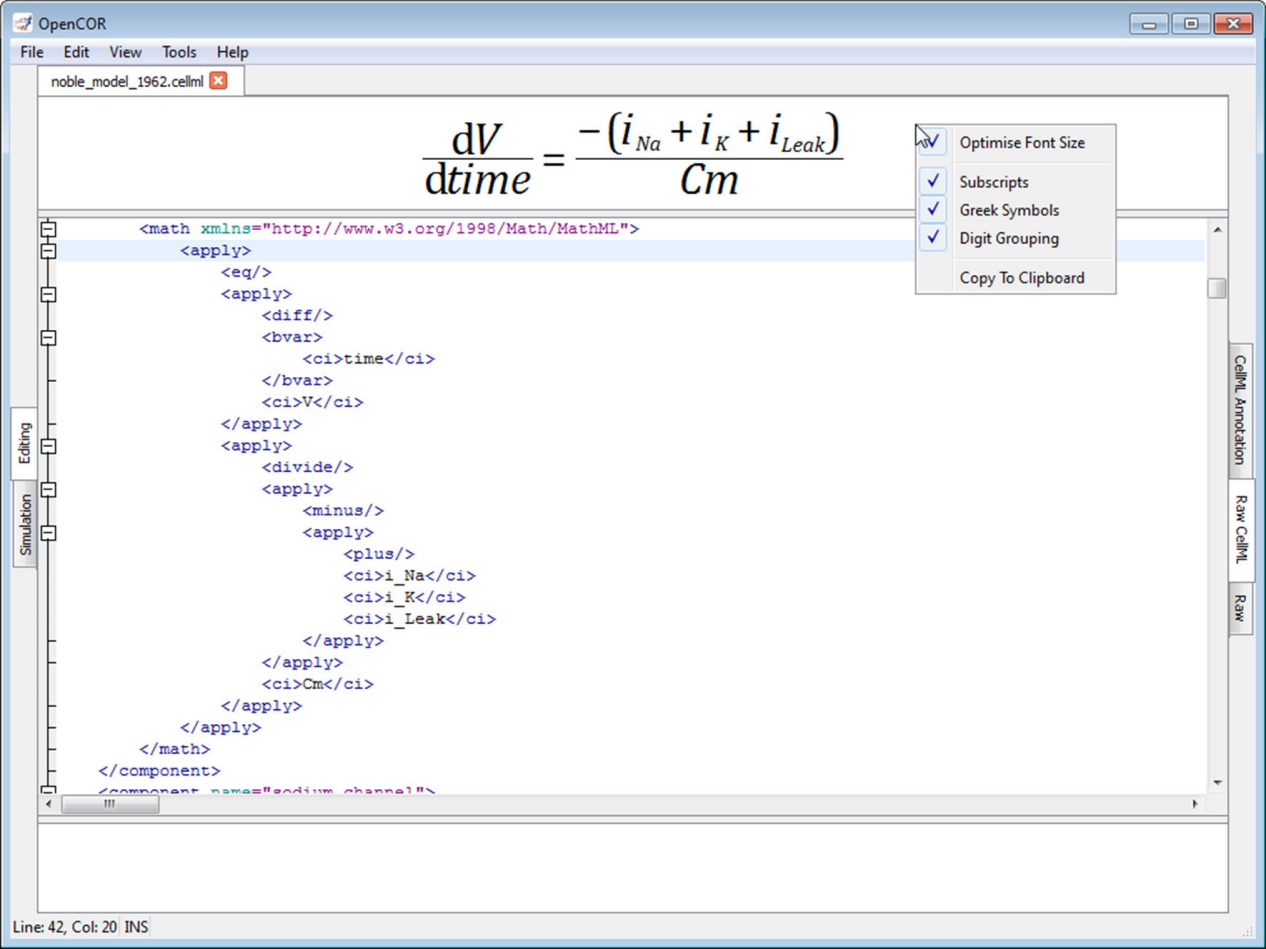
**Raw CellML view**. The *Raw CellML* view builds on the *Raw* view (Figure 5) by adding syntax highlighting for CellML files. For user convenience, mathematical equations are rendered above the editor and in real-time. CellML files can also be validated, resulting in a list of errors/warnings, if needed.

The *Raw CellML view* plugin builds on the *Raw view* plugin (Figure 5) by adding syntax highlighting for CellML files (Figure 6; opencor.ws/user/plugins/editing/RawCellMLView.html). The panel above the editor uses the Qwt library to visualize mathematical equations in real-time, as well as to copy them to the clipboard for use in other programs, if needed. For an equation to be rendered, the editor's caret must be within a valid apply MathML block. If this is not the case, nothing is displayed while a warning sign is displayed if the block is not valid. Different aspects of the panel can be customized: its font size, the grouping of digits, and the rendering of subscripts and Greek symbols. The view can also be used to validate a CellML file. If the CellML file is invalid, a list of errors and/or warnings will be displayed, and to click on any of them will get the editor to jump to the offending line/column.

### Simulation plugin

**Figure 7:**
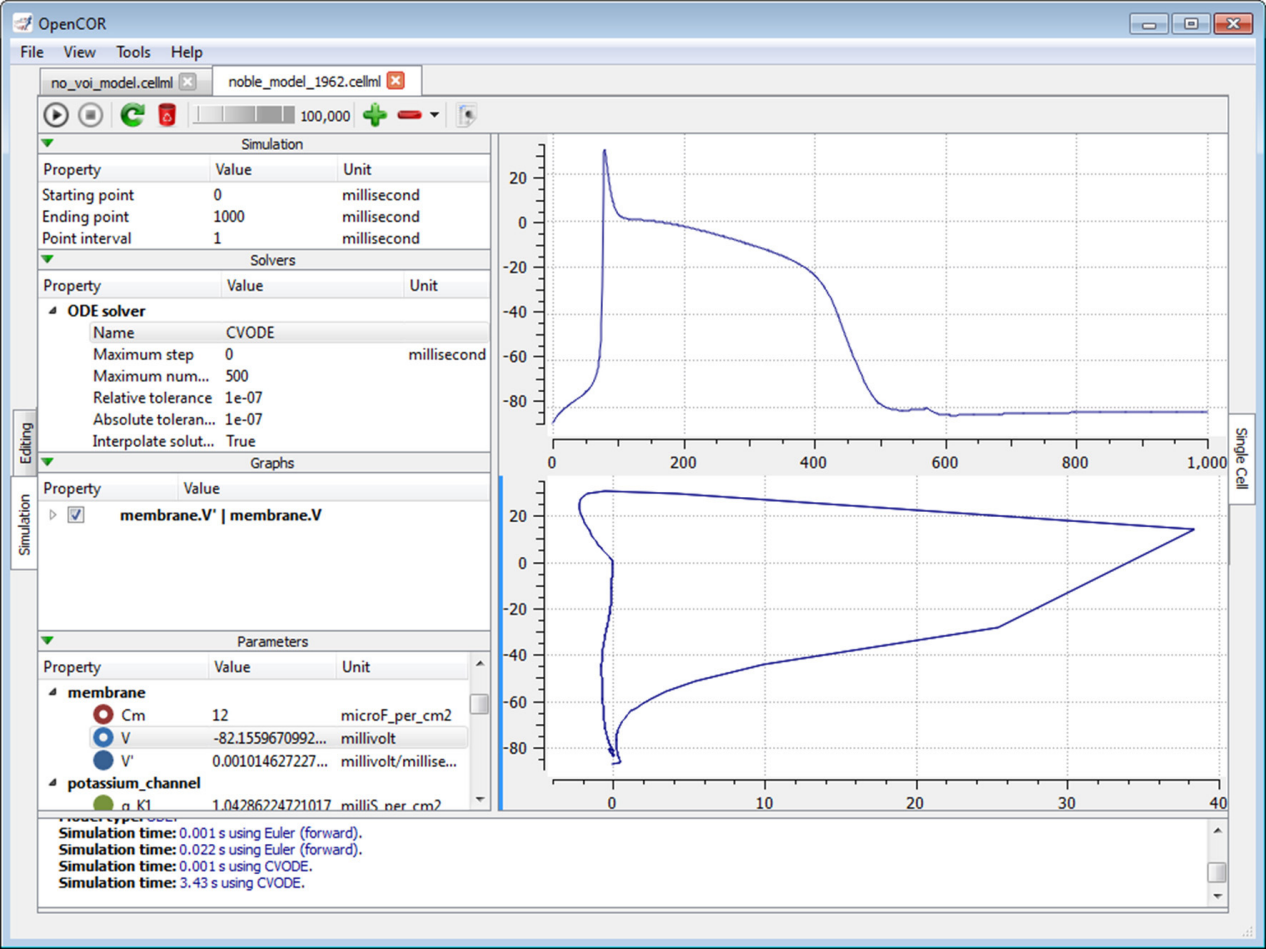
**Single Cell view**. The *Single Cell* view can be used to run CellML files that describe a system of differential equations, be they ordinary or algebraic. Different solvers can be used and any model parameter can be plotted against any other.

The *Simulation* mode currently consists of the *Single Cell view* plugin (Figure 7; opencor.ws/user/plugins/simulation/SingleCellView.html), which can be used to run CellML files that describe a system of ODEs or DAEs. For the view to be usable, it needs a model to be in the form of a runtime. In the case of a CellML file, this first means converting the model to C code using the CellML API. The runtime is then obtained by compiling, on the fly, the C code using LLVM/Clang.

#### Graphical user interface

The view consists of two main parts, the first of which allows the user to customize the simulation, the solver and the model parameters, as well as to create graphs. The second part is used to render the graphs using the Qwt library. All the model parameters listed in the *Parameters* section have an icon associated with them, based on their type: variable of integration (a cyan-filled circle), (editable) constant (a red hollow circle), computed constant (a red-filled circle), (editable) state (a blue hollow circle), rate (a blue-filled circle) or algebraic (a green-filled circle).

#### Running a model

To run a model, the user needs to provide some information about the simulation itself, i.e., its starting point, ending point and point interval. Then, s/he needs to select and, if needed, customize a solver. This done, the model can be run and its simulation data plotted and/or exported to a comma-separated value (CSV) file.

However, this is assuming that there is enough memory to run the model and keep all of its simulation data in memory. This is so that graphs can be added *after* a model has been run, although graphs can be added at any point in time, including prior to running a model. By default, the view has one graph panel with no graphs associated with it, but additional graph panels and graphs can be added, if needed. For example, in Figure 7, there are two graph panels with one graph each.

#### Plotting simulation data

A graph is the result of plotting a model parameter against another. For instance, in the top graph panel of Figure 7, we plotted the parameter V (from the component membrane) against the variable of integration, which here is time. In the bottom graph panel of that same figure, we plotted the same parameter V, but this time against its derivative, i.e., V′, as can be seen under the *Graphs* section.

The layout of the view is independent of the file that is currently selected. This is so that graphs can be created and used with more than just one file and therefore simulation. Looking at Figure 7, we could open and run another CellML file, and the same graphs would be used. That is, as long as that other CellML file has the same variable of integration and parameter V. If it is not the case, then a warning sign will be displayed in front of the graph entry.

A graph can be locked, in which case it will always be linked to a particular CellML file. This feature can, for example, be used to compare the simulation data of two CellML files A and B by having two identical graphs with one of them locked (on, say, A) and by having B selected.

#### Interacting with a simulation

By default, a simulation will run until it reaches its ending point, but it can also be paused and one or several of its parameter values changed, before being resumed. This is what was done in Figure 7 after ~575 ms worth of simulation (see the top graph panel). However, to pause a simulation at a suitable point can be tricky. The view therefore offers a means to artificially slow a simulation down, a feature which can also be useful in a teaching setup.

#### Multithreading

Finally, to keep the GUI responsive, each simulation is run in its own thread. This means that several simulations can also be run in parallel.

### Solver plugins

Solver plugins are of no use on their own, but they can nonetheless be selected (see Subsection Plugins window; Figure 2). This is so that they can, based on the type of a model (i.e., ODE or DAE), be made available to the user when in *Simulation* mode (see Section Simulation plugin).

OpenCOR supports three types of solvers: ODE, DAE, and non-linear algebraic (NLA). The latter type is for ODE and DAE models that require solving one or several systems of NLA equations. To solve such a model, a simulation view like the *Single Cell* view (Figure 7) needs access to an ODE or a DAE solver, as well as to an NLA solver.

Most of the solvers currently shipped with OpenCOR are for solving ODE systems. They implement simple methods such as the Forward Euler method, the Heun's method and the (second- and fourth-order) Runge-Kutta methods. However, more advanced solvers are also available through the SUNDIALS library. They are CVODE, IDA, and KINSOL for ODE, DAE, and NLA systems, respectively.

## Discussion

### Programming languages and frameworks

OpenCOR and its plugins are written in C++ using the Qt framework. In comparison, COR is written in Delphi (Object Pascal) using the VCL framework while OpenCell is written in JavaScript using the XUL framework. Those languages (and others; e.g., Java, Python) and frameworks (and others; e.g., Swing, wxWidgets) have been used or evaluated either by ourselves or some of our close collaborators.

#### Qt framework

The general consensus was that we should use Qt for OpenCOR. Qt is a cross-platform framework that has now been around for nearly 20 years. It is used in some worldwide projects (e.g., KDE, a cross-platform desktop environment), and it can nowadays be fully used under open source license.

#### C++ language

The choice of the C++ language was more subjective, but it was nonetheless made for mainly three reasons. Qt is written in C++, so interaction with Qt is inherently easier in C++ and new Qt releases can be used without delay. The same argument holds true with many third-party libraries, which tend to be written in C/C++ (e.g., LLVM, SUNDIALS). Finally, the overall speed of a compiled language such as C++ is particularly valuable when it comes to the responsiveness of a scientific application like OpenCOR.

#### Python support

However, many people like the flexibility that comes with using Python, in particular when used with libraries such as NumPy (numpy.org) and SciPy (scipy.org). For this reason, we have started work on adding support for Python in OpenCOR, via PythonQt (pythonqt.sourceforge.net). As a proof of concept, we came up with a simple *Python console* window, from which the user can interact with simulation data (e.g., to calculate, using SciPy, an FFT ^4^ from a time series). Ultimately, we will want to be able to interact with various aspects of OpenCOR using Python scripts, as well as have plugins written in Python.

### CellML API

#### “Official” CellML API vs.own CellML API

Neither CellML 1.1 nor the “official” CellML API was released or available when work on COR started. COR therefore had to rely on its own CellML API, which only supports CellML 1.0. In contrast, both OpenCOR and OpenCell use the “official” CellML API and, as such, support both CellML 1.0 and CellML 1.1 “out of the box.”

#### Move to libCellML

However, the “official” CellML API is known to have some limitations (e.g., it may wrongly (in)validate certain CellML files). For those reasons and others, the CellML editorial board (cellml.org/community/editorial_board) decided that a complete rewrite of the API was necessary. So far, no specific timeline has been set for the release of libCellML, the future “official” CellML API. However, CellML support in OpenCOR is done through an intermediate layer that sits between OpenCOR and the CellML API. Support for libCellML should not therefore affect the layer interface, just its implementation.

### Editing Mode

#### CellML annotation capabilities

CellML annotation in OpenCOR consists of annotating any CellML element with one or several ontological terms. For example, we may have Ca_i *isVersionOf* “calcium,” but this is not precise enough. Ideally, we would like to be able to say that Ca_i *is* “concentration” *of* “calcium” *in* “cytosol.” This type of annotation is known as composite annotation and will eventually be supported in OpenCOR through the RICORDO knowledgebase (ricordo.eu; de Bono et al., 2011), as part of our contribution to the Virtual Physiological Rat project (virtualrat.org).

#### CellML authoring capabilities

The *Raw CellML view* plugin allows the editing and validation of CellML files, but to edit raw CellML is neither ideal nor efficient. In COR, CellML authoring is done through an intermediate format, which is a mixture of C and Pascal syntax. Upon loading a CellML file, COR converts the raw CellML code to its intermediate format, for the user to edit. This format is then converted back to raw CellML code for saving on disk. This approach has been well received by both novice and expert users of CellML, with some novice users being able to encode their model without any prior knowledge of CellML. Our intent is to replicate this approach in OpenCOR, starting from the *Raw CellML view* plugin and adding support for COR's intermediate format, to create a *Pretty CellML view* plugin.

### Simulation mode

#### CellML-based approach

CellML files must be processed before the mathematical model they encode can be executed. In OpenCOR, this requires converting a CellML file to C code, which in turn is compiled to get a runtime that is used to execute the encoded model. This is in contrast with the traditional approach where a mathematical model is directly implemented in some programming language. Such an implementation can be optimized by using techniques like partial evaluation and lookup tables (Vigmond et al., 2003), or by targeting GPUs ^5^ (Neic et al., 2012). Similar optimization techniques can also be (automatically, this time) applied to a CellML file (Cooper et al., 2006), but the resulting code will never be as fast as its handcrafted equivalent.

On the other hand, to implement a mathematical model the traditional way is both time-consuming and error prone. Also, the range of modeling studies that can be carried out is limited to the models that have been implemented while, with nearly 600 models, the CellML Model Repository (models.physiomeproject.org/cellml) makes a CellML-based approach very attractive (e.g., Fink et al., 2011).

#### Computational speed

Computational speed is an important consideration when running long and/or complex simulations. So far, COR has proven to be the fastest among various CellML tools (Garny et al., 2008). This result is largely explained by the use, in COR, of advanced computational techniques, as well as by some of COR's own limitations. For example, OpenCOR keeps track of all model parameters while COR only keeps track of those that it plots. Also, OpenCOR relies on LLVM (llvm.org), a generic compiler solution, while COR uses a bespoke compiler. As a consequence, OpenCOR was never going to be as fast as COR. Yet, not only it is well within the same order of magnitude as COR, but it is also faster than OpenCell.

#### Data formats

At the moment, simulation data can be exported to the CSV format. This feature was originally part of the *Single Cell view* plugin. However, support for other formats such as BioSignalML (biosignalml.org; Brooks et al., 2011) would improve the exchange and reuse of biosignals. Therefore, OpenCOR now implements a data store architecture, which can be used by data store plugins to add support for additional data formats. A *CSV data store* plugin has been created, and is now shipped with OpenCOR. A similar data store plugin will also be created for BioSignalML. As for solver plugins, data store plugins are of no use on their own, but they are selectable nonetheless, so that a view plugin like the *Single Cell view* plugin can retrieve them and make them available to users.

### Other standards

OpenCOR's plugin architecture allows for other formats and standards to be supported. SED-ML (sed-ml.org; Waltemath et al., 2011) is one such (XML-based) format. It is used to describe virtual experiments and is therefore critical for reproducible computational biology. For that last reason alone, it is essential that we add SED-ML support to OpenCOR, both to the *Editing* and *Simulation* modes. The *Simulation* mode will have to be extended to the CLI version of OpenCOR, so that SED-ML files can be generated (through Python scripting, for example) and executed in a “batch mode” manner, if needed. Such a feature will also be useful in parameter sweep studies where OpenCOR could be used on facilities such as VPH-Share (vph-share.eu).

### Other CellML tools

OpenCOR and its predecessors, COR and OpenCell, are the only native CellML environments available. There are, however, several tools that offer various levels of support for CellML.

#### “Official” CellML API vs.own CellML API

CSim (code.google.com/p/cellml-simulator), eSolv (esolv.nl) and OpenCMISS (Bradley et al., 2011; physiomeproject.org/software/opencmiss) use the “official” CellML API, which means that they support both versions of CellML “out of the box.” In contrast, tools that use either their own CellML API (like COR; see Subsection “Official” CellML API vs. own CellML API) or their own CellML import mechanism tend to support only CellML 1.0. This is the case, for example, with CESE (cese.sourceforge.net), Chaste (Mirams et al., 2013; www.cs.ox.ac.uk/chaste), JSim (Butterworth et al., 2014; physiome.org/jsim), Myokit (Clerx et al., 2014; myokit.org) and VCell (Moraru et al., 2008; vcell.org).

#### Features of interest

Despite this shortcoming, some of those tools have features worth pointing out. For instance, in the context of reproducible computational biology, Chaste, CSim and Myokit offer attractive alternatives to OpenCOR. Support for reproducibility in CSim is done via SED-ML (see Section Other standards) while in Chaste and Myokit it is done via their own syntax (Cooper et al., 2014).

An important aspect of CellML is that all the quantities used in a model must have units associated with them. The “official” CellML API does support units checking, but it is limited while it is comprehensive in COR, JSim and PyCml (part of Chaste). COR uses PyCml's algorithm and therefore offers better units checking than OpenCOR. However, this will soon change with the release of the *Pretty CellML* view (see Subsection CellML authoring capabilities).

CellML tools traditionally target CPUs ^6^. This is the case with Myokit, but it also targets GPUs using either CUDA (nvidia.com/object/cuda_home_new.html) or OpenCL (khronos.org/opencl). This is a direction that OpenCMISS is also taking (Nickerson et al., 2014), in addition to targeting FPGAs ^7^. It is hoped that this work will be contributed back to the “official” CellML API, which would thus make it possible for OpenCOR to target GPUs and FPGAs too.

Both Chaste and OpenCMISS are numerical libraries that can be used for multiscale modeling. As illustrated in Section Simulation plugin, the focus of OpenCOR is currently on single cell modeling. However, in a similar way to what has been done with the “official” CellML API, it might be possible to make plugins out of those libraries, in which case OpenCOR could also be used for (some simple) multiscale modeling.

## Conclusion

OpenCOR is a cross-platform modeling environment that replaces both COR and OpenCell. It can be freely used by computational biologists to organize, edit, simulate and analyze models that describe a system of ODEs or DAEs encoded in the CellML format.

However, OpenCOR's CellML authoring capabilities still lag behind those of COR, but this issue is being addressed and will soon result in the release of the *Pretty CellML* view, which in turn will result in the official retirement of both COR and OpenCell.

Looking ahead, reproducibility is an important aspect of computational biology and should be addressed in OpenCOR. We will therefore be doing this by adding support for SED-ML in both the *Editing* and *Simulation* modes of OpenCOR.

### Conflict of interest statement

The authors declare that the research was conducted in the absence of any commercial or financial relationships that could be construed as a potential conflict of interest.

## Footnotes

^1^API, Application Programming Interface.

^2^URI, Uniform Resource Identifier.

^3^URN, Uniform Resource Name.

^4^FFT, Fast Fourier Transform.

^5^GPU, Graphics Processing Unit.

^6^CPU, Central Processing Unit.

^7^FPGA, Field Programmable Gate Array.
